# Risk Assessment of Liver Metastasis in Pancreatic Cancer Patients Using Multiple Models Based on Machine Learning: A Large Population-Based Study

**DOI:** 10.1155/2022/1586074

**Published:** 2022-05-18

**Authors:** Qinggang Li, Lu Bai, Jiyuan Xing, Xiaorui Liu, Dan Liu, Xiaobo Hu

**Affiliations:** ^1^Department of Infectious Diseases, The First Affiliated Hospital of Zhengzhou University, Zhengzhou, 450052 Henan, China; ^2^Department of Gastroenterology, The First Affiliated Hospital of Zhengzhou University, Zhengzhou, 450052 Henan, China

## Abstract

**Background:**

A more accurate prediction of liver metastasis (LM) in pancreatic cancer (PC) would help improve clinical therapeutic effects and follow-up strategies for the management of this disease. This study was to assess various prediction models to evaluate the risk of LM based on machine learning algorithms.

**Methods:**

We retrospectively reviewed clinicopathological characteristics of PC patients from the Surveillance, Epidemiology, and End Results database from 2010 to 2018. The logistic regression, extreme gradient boosting, support vector, random forest (RF), and deep neural network machine algorithms were used to establish models to predict the risk of LM in PC patients. Specificity, sensitivity, and receiver operating characteristic (ROC) curves were used to determine the discriminatory capacity of the prediction models.

**Results:**

A total of 47,919 PC patients were identified; 15,909 (33.2%) of which developed LM. After iterative filtering, a total of nine features were included to establish the risk model for LM based on machine learning. The RF showed the most promising results in the prediction of complications among the models (ROC 0.871 for training and 0.832 for test sets). In risk stratification analysis, the LM rate and 5-year cancer-specific survival (CSS) in the high-risk group were worse than those in the intermediate- and low-risk groups. Surgery, radiotherapy, and chemotherapy were found to significantly improve the CSS in the high- and intermediate-risk groups.

**Conclusion:**

In this study, the RF model constructed could accurately predict the risk of LM in PC patients, which has the potential to provide clinicians with more personalized clinical decision-making recommendations.

## 1. Introduction

Pancreatic cancer (PC) is the fourth leading cause of cancer-related mortality in the USA, and it causes an estimated 25,270 deaths per year worldwide, accounting for 8% of the total cancer death toll [1]. Pancreatic cancer has a 5-year survival rate of <8%, and up to 80% of patients with PC already have distant organ metastasis at the time of diagnosis, which significantly reduces survival benefits from surgical resection of the primary tumor [2]. Thus, an accurate assessment of locoregional and/or distant metastases in patients with PC is essential to determine whether these patients should undergo additional surgical resection or other combination therapies.

The liver is the most common metastasis site, accounting for 37-41.9% of the initially diagnosed cases [3, 4]. Moreover, more than 60% of the patients that undergo tumor resection relapse with distant liver recurrence within the first 24 months after surgery [5]. Magnetic resonance imaging, computed tomography, and ultrasonography are currently the most commonly used inspection methods. Restricted by economics, doctors' ability, and other aspects, this will affect the judgment of clinicians to a significant extent. Thus, a better prognostic model for the prediction of liver metastasis (LM) in PC is critical to improve treatment and patient outcomes.

The dismal outcomes of PC partly result from its aggressive metastatic nature, but applying appropriate treatment options according to different disease processes can improve the survival rate of patients. In this study, we plan to establish a novel prediction model for liver metastasis based on clinical parameters and simple histopathological with high reliability, which could help to improve patient risk stratification in early PC.

## 2. Materials and Methods

### 2.1. Data Source and Study Population

This retrospective study was carried out based on the Surveillance, Epidemiology, and End Results (SEER) database. The publicly available data was collected from 18 cancer registries between January 1, 2010, and December 31, 2018, using SEER-Stat software (ver. 8.3.5). The patients' files from the SEER database were accessed with official permission, and patients' records were anonymized. The study was approved by the Ethics Committee of the National Cancer Center/National Clinical Research Center for Cancer/Cancer Hospital, Chinese Academy of Medical Sciences.

### 2.2. Main Outcomes and Selected Variables

**Figure 1:**
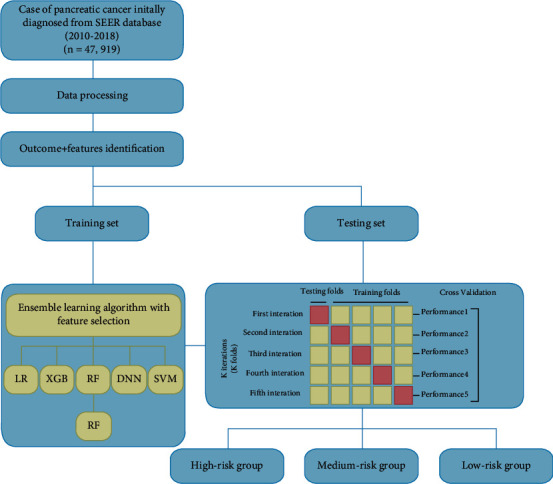
Flow diagram of the study population. A total of 47,919 pancreatic cancer patients were included in this study, which were divided into independent training and independent testing sets in a ratio of 8 : 2. The predictive models and risk stratification were established to help to provide reliable individual information for PC treatment recommendations. Abbreviations: SEER: Surveillance, Epidemiology, and End Results; LR: logistic regression; XGboost: extreme gradient boosting; RF: random forest; DNN: deep neural network; SVM: support vector machine; PC: pancreatic cancer.

Patients with primary pancreatic cancer were included in this cohort study. The target outcome was hepatic metastasis of pancreatic cancer. The cancer diagnosis was based on the classification of the topography or histology based on the International Classification of Diseases for Oncology-3 (ICD-O-3)/WHO 2008 guidelines. The primary pancreatic tumor locations included C25.0—head of the pancreas, C25.1—body of the pancreas, C25.2—tail of the pancreas, C25.3—pancreatic duct, C25.4—islets of Langerhans, C25.7—other specified parts of the pancreas, C25.8—overlapping lesion of the pancreas, C25.9—pancreas, and NOS (not otherwise specified). The exclusion criteria were as follows: (1) the presence or absence of metastasis at diagnosis was unknown; (2) pancreatic cancer patients without pathohistological diagnosis; (3) patients younger than 20 years; (4) patients with benign or borderline tumors; and (5) patients with lacking information on race, histological type, and treatment strategy. The derived American Joint Committee on Cancer (AJCC) 6th and SEER combined stage (2016+) TNM staging was used in this study. Patient demographics included gender, age, year at initial diagnosis, and race. Tumor characteristics included lymph biopsy, surgery, tumor size, marital status, survival status, survival time, the presence of distant metastasis, TNM staging (tumor, lymph node metastasis, and distant metastasis), insurance status, and radiation and chemotherapy records. A flowchart of the data collection process is presented in Figure 1.

### 2.3. Feature Engineering and Data Transformation

These readily available clinical and demographic variables from SEER database were processed to establish the available models using feature engineering techniques. According to the clinical characteristic or median, the continuous variables (age and year at initial diagnosis, tumor size, and number of positive lymph biopsies) were converted into categorical variable. To promote the availability of the prediction model, we employed cross-validation (CV) and recursive feature elimination to iteratively filter variables using the random forest (RF) classifier. CV was used for internal validation as a robust method for evaluating the progress of machine learning and improve the model performance [6]. The variables were evaluated based on their relative importance for the receiver operating characteristic (ROC) of the models.

### 2.4. Risk Model Establishment and Risk Stratification

All of the patients included in this study were randomly divided into independent training (80%) and testing (20%) sets using R [7]. The prediction models were built based on the training sets, after which they were evaluated and validated based on the test set. The extreme gradient boosting (XGboost), RF, SVM [8], deep neural network (DNN), and logistic regression (LR) algorithms were trained by performing 10-fold CV on the training set. Univariate and multivariate logistic regression analyses were employed to evaluate the features significantly correlated with the risk of hepatic metastasis. In addition, correction analysis was performed on features included in this study to evaluate their mutual relationships. The machine learning models were established and evaluated using the caret package in R.

According to our preliminary findings, performance of these different machine learning algorithms was roughly the same for predicting LM, but there was a trend toward improved availability for RF on both training and testing sets. To further evaluate the risk of HM for PC patients, we calculated the risk scores for every patient based on the RF and then sorted the patients based on the risk scores form high to low. The pancreatic cancer patients were divided into three risk group of the same number: high-risk group, intermediate-risk group, and low-risk group, which can inform the selection of a suitable treatment strategy [9].

### 2.5. Statistical Analysis

The chi-squared test was employed to assess the significance of differences among categorical variables in the training set and test set, while the Mann–Whitney *U* test was used for continuous variables. The Kaplan-Meier method and log-rank test were used to evaluate the differences among different subgroups in univariate survival analysis. The cancer-specific survival (CSS) and the survival time were the main evaluation indices. Propensity score matching (PSM) was used to balance the patients at a ratio of 1 : 1 between PC with and without treatment. To measure the performance of several models, the sensitivity, specificity, Gini, and area under the ROC curve, as well as the 95% confidence intervals (CIs) were calculated based on the number of correctly classified TP (true positive) cases and the number of the incorrectly classified FP (false positive) cases. The DeLong test was employed to evaluate model performance in identifying liver metastasis (*P* < 0.05). All analyses were performed using R version 3.6.1.

## 3. Results

### 3.1. Demographic and Clinicopathological Characteristics

A total of 47,919 pancreatic cancer patients from SEER database were analyzed in this study (Figure 1). Of whom, 15,909 (33.2%) patients have developed liver metastasis. 20,046 (41.8%) PC patients were over 70 years old, 30,702 (64.1%) were in T3/T4 stage, and 19,313 (40.3%) were in N1/N2 stage. The more PC patients suffered the tumor in head (39.0%) and tail (24.1%) than in body (18.0%) of the pancreas developed the LM. All of the patients were randomly divided into the training set (*n* = 38,336) and an internal test set (*n* = 9, 583) with the ration of 8 : 2 (Figure 1). All demographic and clinicopathological variables of these patients are detailed in Table 1.

### 3.2. Variable Feature Importance of Liver Metastasis Prediction

To evaluate the association between these features and the risk of liver metastasis, the univariate and multivariate logistic regression was performed for linear correlation analysis (Table 2). The results showed that the age at PC diagnosis, gender, race, primary tumor site, T and N stage, tumor histology, size, surgery performed, chemotherapy, and radiotherapy were significant prognosis factors for predicting liver metastasis in univariate and multivariate logistic regression analysis (*P* < 0.05). And the tumor in the body (adjusted OR, 1.63; 95CI, 1.53-1.73; *P* < 0.001) and tail (adjusted OR, 3.23; 95CI, 3.02-3.45; *P* < 0.001) of the pancreas suffered higher risk for liver metastasis than in the head of the pancreas. Both chemotherapy (adjusted OR, 0.17; 95CI, 0.16-0.19; *P* < 0.001) and surgery (adjusted OR, 0.10; 95CI, 0.09-0.11; *P* < 0.001) performed could significantly decrease the risk of liver metastasis for pancreatic cancer. But radiotherapy (adjusted OR, 1.08; 95CI, 1.03-1.13; *P* < 0.001) was positively related with the risk of liver metastasis.

### 3.3. Model Performance

To establish the available predicting models, we used recursive feature elimination and 10-fold-CV to iteratively select features based on the implementation of the RF classifier. Besides, nine features (tumor histology, chemotherapy, N stage, age at PC diagnosis, tumor size, primary tumor site, T stage, radiotherapy, and surgery) were selected and included in machine learning development.

**Figure 2:**
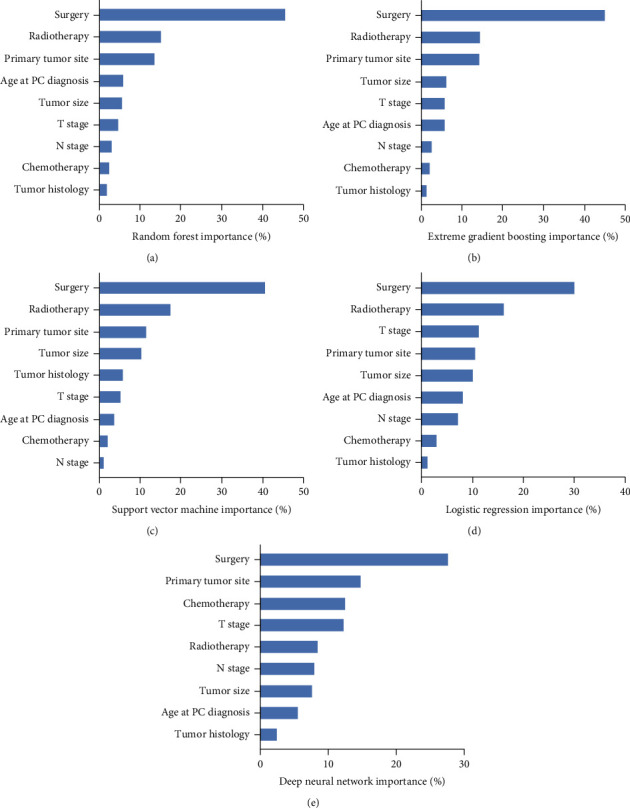
The feature importance for predicting liver metastasis in diverse predictive machine models. The feature importance (a) for random forest, (b) for extreme gradient boosting, (c) for support vector machine, (d) for logistic regression, and (e) for deep neural network. Abbreviations: PC: pancreatic cancer.

Five risk models were established based on the selected features. We evaluated the importance of selected features by the size of the gain value for predicting liver metastasis in five models (Figure 2). Although the importance of features varied slightly among different models, the overall results noted that surgery, radiotherapy, primary tumor site, and tumor size ranked at the top of the list. The tumor treatments (including surgery, radiotherapy, and chemotherapy) were associated closely with liver metastasis.

The specificity, sensitivity, ROC value, and Gini scores were constructed to identify the reliability of model (Table 3). The results showed that the RF model had the best performance in both training and test sets (ROC = 0.871 and 0.832, respectively), compared with XGB (ROC = 0.838 and 0.837, respectively), DNN (ROC = 0.830 and 0.832, respectively), SVM (ROC = 0.813 and 0.839, respectively), and LR (ROC = 0.817 and 0.821, respectively). The sensitivity and specificity values of the predictions noted the same results.

### 3.4. Risk Stratification for Patients

**Figure 3:**
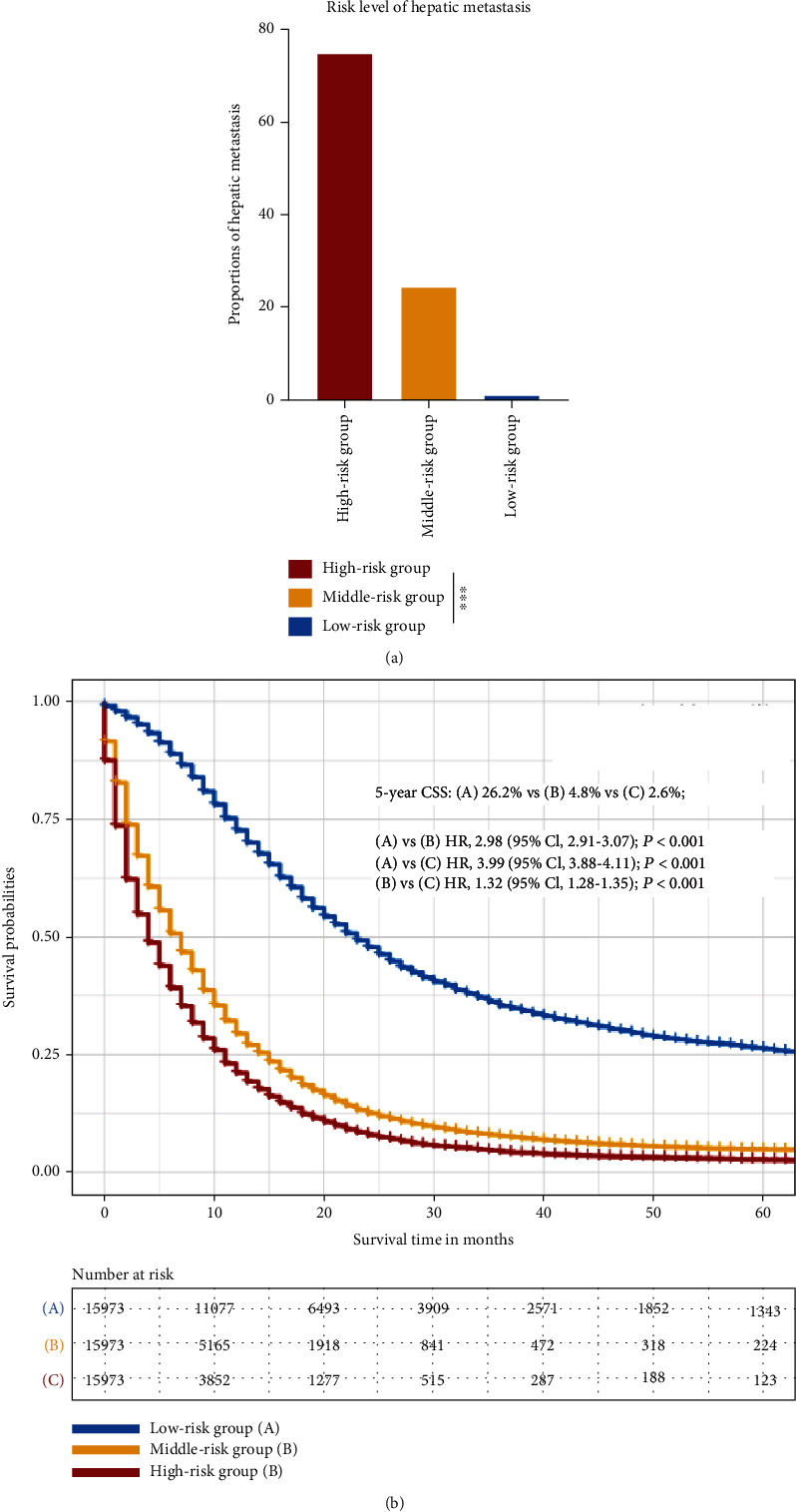
Risk levels for predicting liver metastasis in pancreatic cancer by using random forest. (a) The risk scores of developing liver metastasis based on random forest. Sorted by risk scores form high to low, pancreatic cancer patients were divided into three risk group of the same number: high-risk group, middle-risk group, and low-risk group. The liver metastasis rates were significantly higher in the high-risk group than the others (^∗∗∗^*P* < 0.001). (b) The survival comparison among three risk different group. The CSS were significantly worse than the others. Abbreviations: CSS: cancer-specific survival; HR: hazard ratio; CI: confidence interval.

We calculated the risk score for pancreatic cancer patients for predicting liver metastasis with RF classifier. These PC patients were assigned to an average of three risk groups according to their risk scores ranked from high to low and about 15,973 (33.3%) patients in every risk group (Figure 3(a)); the patients had the highest risk scores in the high-risk group and the lowest in the low-risk group. The result on proportions of liver metastasis showed 11,905 (74.5%) patients with liver metastasis in the high-risk group, 3898 (24.4%) patients in the middle-risk group, and 106 (0.7%) patients in the low-risk group. There was significant difference of proportions of liver metastasis among three groups (*P* < 0.001). And then, we compare the pancreatic cancer 5-year CSS among the three groups (Figure 3(b)); the survival probabilities were significantly different among three groups; the 5-year CSS was 2.6% in the high-risk group, 4.8% in the middle-risk group, and 26.2% in the low-risk group. The univariate Cox regression analysis noted that low-risk group vs. middle-risk group was HR, 2.98; 95CI, 2.91-3.07; *P* < 0.001; low-risk group vs. high-risk group was HR, 3.99; 95CI, 3.88-4.11; *P* < 0.001; and middle-risk group vs. middle-risk group was HR, 1.32; 95CI, 1.28-1.35; *P* < 0.001; the pancreatic cancer patients with higher risk scores had worse survival.

### 3.5. The Treatment for Three Risk Groups

**Figure 4:**
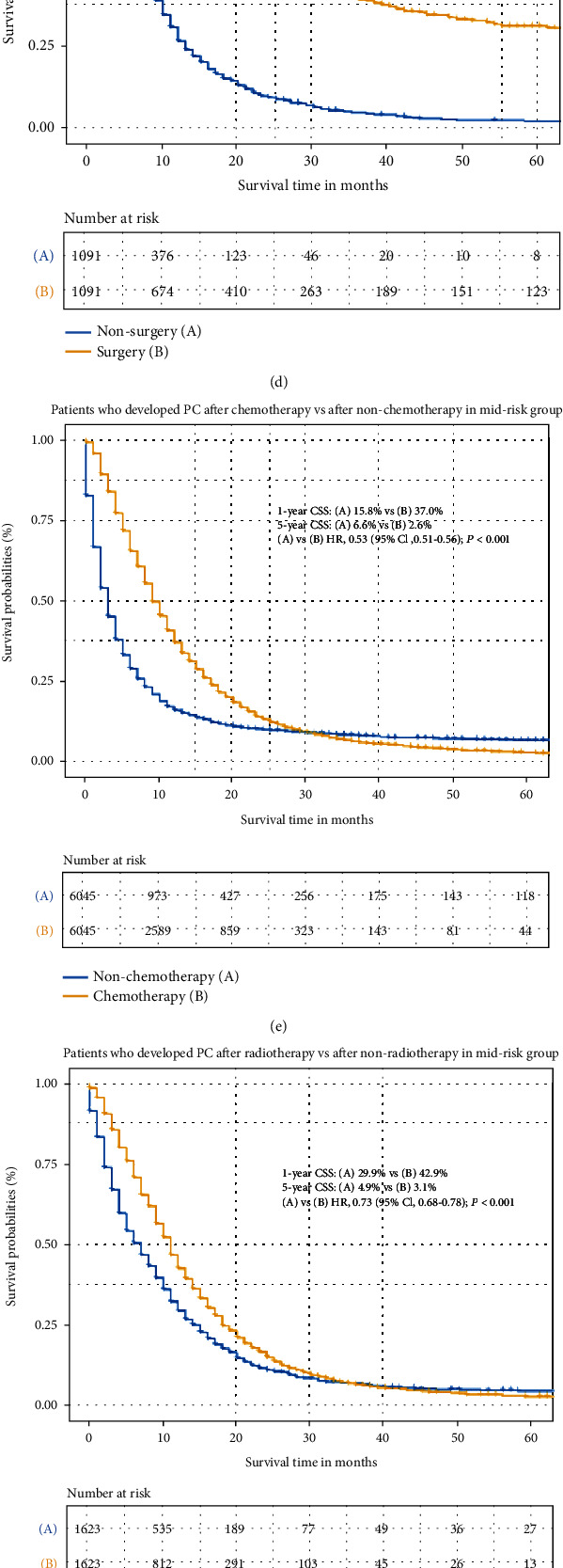
(a) The cancer-specific survival (CSS) comparison between pancreatic cancer (PC) patients who receive surgery and nonsurgery in the high-risk group (after PSM). (b) Survival comparison between PC patients who receive chemotherapy and nonchemotherapy in the high-risk group (after PSM). (c) Survival comparison between PC patients who receive radiotherapy and nonradiotherapy in the high-risk group (after PSM). (d) Survival comparison between pancreatic cancer (PC) patients who receive surgery and nonsurgery in the middle-risk group (after PSM). (e) Survival comparison between PC patients who receive chemotherapy and nonchemotherapy in the middle-risk group (after PSM). (f) Survival comparison between PC patients who receive radiotherapy and nonradiotherapy in the middle-risk group (after PSM). (g) Survival comparison between pancreatic cancer (PC) patients who receive surgery and nonsurgery in the low-risk group (after PSM). (h) Survival comparison between PC patients who receive chemotherapy and nonchemotherapy in the low-risk group (after PSM). (i) Survival comparison between PC patients who receive radiotherapy and nonradiotherapy in the low-risk group (after PSM). Note: the PC patients who receive treatment (including surgery, chemotherapy, and radiotherapy) and nontreatment at a PSM ratio of 1 : 1. (a, d, g) PC patients who receive surgery and PC patients who did not receive surgery were matched by PSM at a ratio of 1 : 1. (b, e, h) PC patients who receive chemotherapy and PC patients who did not receive chemotherapy were matched by PSM at a ratio of 1 : 1. (c, f, i) PC patients who receive radiotherapy and PC patients who did not receive radiotherapy were matched by PSM at a ratio of 1 : 1. The matched variables for propensity score matching (PSM) included the age at PC diagnosis, race, gender, T stage, N stage, year of PC diagnosis, tumor size, and histology at the ratio of 1 : 1. Abbreviations: CSS: cancer-specific survival; HR: hazard ratio; CI: confidence interval; PSM: propensity score matching; PC: pancreatic cancer.

To evaluate the therapeutic effect of performed surgery, chemotherapy, and radiotherapy for pancreatic cancer patients in different risk score groups, we balanced the demographic and clinicopathological characteristics of patients receiving or nonreceiving treatment with propensity score matching based on the age at PC diagnosis, race, gender, T stage, N stage, year of PC diagnosis, tumor size, and histology at the ratio of 1 : 1 between patients receiving and not receiving performed surgery, chemotherapy, or radiotherapy. And we analyzed the 1-year and 5-year CSS for patients with balanced baseline in different risk groups. In the high-risk group, the patients receiving surgery (HR, 0.31; 95CI, 0.21-0.46; *P* < 0.001), chemotherapy (HR, 0.42; 95CI, 0.40-0.44; *P* < 0.001), and radiotherapy (HR, 0.81; 95CI, 0.69-0.96; *P* = 0.012) had better CSS than patients not receiving treatment (Figures 4(a)–4(c)). In the middle-risk group, the patients receiving surgery (HR, 0.31; 95CI, 0.28-0.35; *P* < 0.001), chemotherapy (HR, 0.53; 95CI, 0.51-0.56; *P* < 0.001), and radiotherapy (HR, 0.72; 95CI, 0.60-0.78; *P* < 0.001) had better CSS than patients not receiving treatment (Figures 4(d)–4(f)). In the low-risk group, the patients receiving surgery (HR, 0.29; 95CI, 0.27-0.32; *P* < 0.001) had better survival than patients with nonsurgery (Figure 4(g)). But receiving chemotherapy and radiotherapy may not promote the survival and prognosis for pancreatic cancer patients in the low-risk group (Figures 4(h) and 4(i)).

## 4. Discussion

In this study, we collected data from the SEER database, which covers 47,919 patients with PC. The trends in this dataset are therefore highly representative and universal. We described the clinical characteristics of PC patients with or without LM and factors that predict the risk of LM in these patients. The univariate and multivariate logistic regression analyses showed that the age at PC diagnosis, gender, race, primary tumor site, T and N stage, tumor histology, size, surgery, chemotherapy, and radiotherapy were significantly correlated with the risk of liver metastasis in PC. This result was consistent with similar studies. Compared with elderly patients, metastases are more often observed in younger patients, who usually have more malignant tumors with more aggressive histological features, which may lead to higher rates of liver metastasis or other forms of distant metastasis [10, 11]. Gender is related to liver metastases, which are less frequent in female patients [12]. Tumor site, grade, size, and LN metastasis were all previously identified as independent predictors of liver metastasis in patients with PC [13]. Studies have shown that primary tumors located in the body and tail of the pancreas are more prone to liver metastases than primary tumors that occur in the head of the pancreas. Compared with tumors located in the head of the pancreas, PC in the body and tail is larger or more frequently diagnosed at an advanced stage, which may increase the risk of liver metastases in these patients [14]. Since patient counseling and decision are based on the estimated from the individual risk profiles, these risk factors may help customize liver monitoring and clinical decision-making.

Distant metastasis is a sign of advanced cancer, indicating a poor prognosis for PC patients. Approximately 60% of pancreatic cancer patients are diagnosed with metastasis, especially liver metastasis [15]. Surgery is considered to be the best potential curative treatment for PC patients, but the indications for tumor resection remain controversial. Although a few scholars disagree [16, 17], most studies advocate that surgical resection of the primary tumor and liver metastases should be the preferred choice for patients with resectable PC with liver metastases [18–20]. Surgical removal of the primary tumor and metastases can improve the quality of life and prolong survival, especially in patients with oligometastatic PC [21–23]. Timely diagnosis of LM is therefore crucial, since it can provide evidence and recommendations for oncologists to make appropriate clinical treatment decisions. Unfortunately, conventional imaging tests for the diagnosis of liver metastases such as Doppler ultrasound, magnetic resonance imaging, or computed tomography have not shown high sensitivity and specificity in PC [24, 25]. Moreover, multiple imaging examinations will also increase the financial burden of patients. Therefore, it is important to establish a model that can accurately predict the probability of LM in PC patients. In this study, we assessed available predictive models using the SEER dataset, which demonstrates significant discrimination and calibration and can provide a basis for formulating an optimal surgical plan. Using this approach, PC patients can be divided into different risk grades to formulate different LM review plans according to the level of risk. Effective clinical decision-making can save large amounts of time and economic costs for patients.

In spite of its promising results, this study still has several limitations. First, this is a retrospective study. Second, due to intrinsic limitations of the database, nonunified selection criteria were employed for patients and detailed information about the treatment was not recorded, such as operation details, chemotherapy plan, and radiation therapy plan, inter alia. Third, the major limitation of our study is the lack of important variables, such as time-to-treatment, type of surgery, patient status, and tumor burden at the surgical margin. Finally, further validation based on a large-scale external cohort is needed.

## 5. Conclusion

The RF model constructed in this study could accurately predict the risk of LM in PC patients, which may provide clinicians with more personalized clinical decision-making recommendations. The therapeutic effect of treatment is expected to be different for pancreatic cancer patients in the three risk groups based on the RF model. Machine learning technology has the potential to provide reliable individual PC treatment recommendations.

## Figures and Tables

**Table 1:** Demographic and tumor characteristics of pancreatic cancer patients.

Characteristic	All patients		Training set		Test set	
	None (*n* = 32,010)	Liver metastasis (*n* = 15,909)	None (*n* = 25,525)	Liver metastasis (*n* = 12,811)	None (*n* = 6485)	Liver metastasis (*n* = 3098)
Age at PC diagnosis, no. (%) (years)						
20-49	2175 (6.8)	1143 (7.2)	1701 (6.7)	945 (7.4)	474 (7.3)	198 (6.4)
50-69	15,834 (49.5)	8721 (54.8)	12,606 (49.4)	7064 (55.1)	3228 (49.8)	1657 (53.5)
≥70	14,001 (43.7)	6045 (38.0)	11,218 (43.9)	4802 (37.5)	2783 (42.9)	1243 (40.1)
Gender, no. (%)						
Female	15,996 (49.9)	7128 (44.8)	12,734 (49.9)	5728 (44.7)	3262 (50.3)	1400 (45.2)
Male	16,014 (50.1)	8781 (55.2)	12,791 (50.1)	7083 (55.3)	3223 (49.7)	1698 (54.8)
Year of PC diagnosis, no. (%)						
2010-2013	12,797 (40.0)	6086 (38.3)	10,207 (40.0)	4912 (38.3)	2590 (39.9)	1174 (37.9)
2014-2018	19,213 (60.0)	9823 (61.7)	15,318 (60.0)	7899 (61.7)	3895 (60.1)	1924 (62.1)
Race, no. (%)						
White	25,194 (78.7)	12,460 (78.3)	20,079 (78.7)	10,052 (78.5)	5115 (78.9)	2408 (77.7)
Black	3823 (11.9)	2204 (13.9)	3063 (12.0)	1765 (13.8)	760 (11.7)	439 (14.2)
Other	2993 (9.4)	1245 (7.8)	2383 (9.3)	994 (7.8)	610 (9.4)	251 (8.1)
Primary tumor site, no. (%)						
Head of the pancreas	20,040 (62.6)	6205 (39.0)	15,925 (62.4)	4951 (38.6)	4115 (63.5)	1254 (40.5)
Body of the pancreas	4177 (13.0)	2859 (18.0)	3399 (13.3)	2341 (18.3)	778 (12.0)	518 (16.7)
Tail of the pancreas	3546 (11.1)	3834 (24.1)	2805 (11.0)	3115 (24.3)	741 (11.4)	719 (23.2)
Overlapping lesion of the pancreas, no. (%)	4247 (13.3)	3011 (18.9)	3396 (13.3)	2404 (18.8)	851 (13.1)	607 (19.6)
AJCC T stage						
T1/T2	10,010 (31.3)	7507 (45.3)	8001 (31.3)	5771 (45.0)	2009 (31.0)	1436 (46.4)
T3/T4	22,000 (68.7)	8702 (54.7)	17,524 (68.7)	7040 (55.0)	4476 (69.0)	1662 (53.6)
AJCC N stage, no. (%)						
N0	18,803 (58.7)	9803 (61.6)	15,010 (58.8)	7901 (61.7)	3793 (58.5)	1902 (61.4)
N1/N2	13,207 (41.3)	6106 (38.4)	10,515 (41.2)	4910 (38.3)	2692 (41.5)	1196 (38.6)
Tumor histology, no. (%)						
Adenocarcinomas	22,944 (71.7)	13,542 (85.1)	18,342 (71.9)	10,874 (84.9)	4602 (71.0)	2668 (86.1)
Other	9066 (28.3)	2367 (14.9)	7183 (28.1)	1937 (15.1)	1883 (29.0)	430 (13.9)
Tumor size, no. (%) (cm)						
0-2	3956 (12.4)	839 (5.3)	3177 (12.4)	665 (5.2)	779 (12.0)	174 (5.6)
2-5	17,325 (54.1)	6789 (42.7)	13,779 (54.0)	5475 (42.7)	3546 (54.7)	1314 (42.4)
>5	10,729 (33.5)	8281 (52.1)	8569 (33.6)	6671 (52.1)	2160 (33.3)	1610 (52.0)
Number of nodes examined, no. (%)						
None	17,918 (56.0)	14,865 (93.4)	14,332 (56.1)	11,945 (93.2)	3586 (55.3)	2920 (94.3)
One or more	14,092 (44.0)	1044 (6.6)	11,193 (43.9)	866 (6.8)	2899 (44.7)	178 (5.7)
Surgery, no. (%)						
No	18,580 (58.0)	15,339 (96.4)	14,821 (58.1)	12,336 (96.3)	3759 (58.0)	3002 (96.9)
Yes	13,430 (42.0)	571 (3.6)	10,704 (41.9)	475 (3.7)	2726 (42.0)	96 (3.1)
Chemotherapy, no. (%)						
No	12,164 (38.0)	6576 (41.3)	9744 (38.2)	5272 (41.2)	2420 (37.3)	1302 (42.1)
Yes	19,846 (62.0)	9333 (58.7)	15,781 (61.8)	7539 (58.8)	4065 (62.7)	1794 (57.9)
Radiation, no. (%)						
No	24,096 (75.3)	15,174 (95.4)	19,233 (75.3)	12,201 (95.2)	4863 (75.0)	2973 (96.0)
Yes	7914 (24.7)	735 (4.6)	6292 (24.7)	610 (4.8)	1622 (25.0)	125 (4.0)

Note: all of the patients included in this study were randomly divided into independent training and independent testing sets in a ratio of 8 : 2. Abbreviations: AJCC: American Joint Committee on Cancer; PC: pancreatic cancer.

**Table 2:** Univariate and multivariate logistic regression to identify risk factors related to liver metastasis for pancreatic cancer patients.

Characteristic	Univariate logistic regression		Multivariate logistic regression	
	HR (95CI)	*P* value	HR (95CI)	*P* value
Age at PC diagnosis				
20-49	Ref		Ref	
50-69	1.05 (0.97-1.13)	0.227	0.75 (0.68-0.82)	<0.001
≥70	0.82 (0.76-0.89)	<0.001	0.48 (0.43-0.53)	<0.001
Gender				
Female	Ref		Ref	
Male	1.23 (1.18-1.28)	<0.001	1.15 (1.1-1.2)	<0.001
Year of PC diagnosis				
2010-2013	Ref		Ref	
2014-2018	1.08 (1.03-1.12)	<0.001	0.98 (0.93-1.03)	0.358
Race, no. (%)				
White	Ref		Ref	
Black	1.17 (1.1-1.23)	<0.001	1.02 (0.95-1.09)	0.58
Other	0.84 (0.78-0.9)	<0.001	0.88 (0.81-0.96)	0.002
Primary tumor site				
Head of the pancreas	Ref		Ref	
Body of the pancreas	2.21 (2.09-2.34)	<0.001	1.63 (1.53-1.73)	<0.001
Tail of the pancreas	3.49 (3.31-3.69)	<0.001	3.23 (3.02-3.45)	<0.001
Overlapping lesion of the pancreas	2.29 (2.17-2.42)	<0.001	1.63 (1.53-1.74)	<0.001
AJCC T stage				
T1/T2	Ref		Ref	
T3/T4	1.55 (1.53-1.57)	<0.001	1.5 (1.47-1.52)	<0.001
AJCC N stage				
N0	Ref		Ref	
N1/N2	1.89 (1.85-1.92)	<0.001	1.64 (1.56-1.73)	<0.001
Tumor histology				
Adenocarcinomas	Ref		Ref	
Other	0.44 (0.42-0.47)	<0.001	0.83 (0.78-0.88)	<0.001
Tumor size				
0-2	Ref		Ref	
2-5	1.85 (1.71-2)	<0.001	1.69 (1.54-1.85)	<0.001
>5	3.64 (3.36-3.94)	<0.001	2.64 (2.4-2.9)	<0.001
Number of nodes examined				
None	Ref		Ref	
One or more	0.09 (0.08-0.1)	<0.001	0.44 (0.39-0.48)	<0.001
Surgery				
No	Ref		Ref	
Yes	0.05 (0.05-0.06)	<0.001	0.1 (0.09-0.11)	<0.001
Chemotherapy				
No	Ref		Ref	
Yes	0.87 (0.84-0.9)	<0.001	0.17 (0.16-0.19)	<0.001
Radiotherapy				
No	Ref		Ref	
Yes	0.15 (0.14-0.16)	<0.001	1.08 (1.03-1.13)	<0.001

Note: the univariate and multivariate logistic regression was established in all patients set. Abbreviations: AJCC: American Joint Committee on Cancer; PC: pancreatic cancer.

**Table 3:** Performance of machine learning model.

Prediction models	Training set					Test set				
	ROC		Gini index	Specificity	Sensitivity	ROC		Gini index	Specificity	Sensitivity
Random forest	0.871	(0.868-0.874)	0.742	0.718	0.854	0.832	(0.824-0.840)	0.664	0.665	0.875
Extreme gradient boosting	0.838	(0.833-0.841)	0.676	0.665	0.854	0.837	(0.829-0.845)	0.674	0.671	0.860
Deep neural network	0.830	(0.826-0.835)	0.660	0.681	0.827	0.832	(0.824-0.840)	0.664	0.619	0.900
Support vector machine	0.813	(0.808-0.817)	0.626	0.672	0.839	0.812	(0.803-0.821)	0.624	0.696	0.826
Logistic regression	0.817	(0.813-0.822)	0.634	0.702	0.791	0.821	(0.813-0.829)	0.642	0.689	0.814

## Data Availability

Corresponding authors may provide data to support the findings of this study upon reasonable request.

## Supplementary Materials

Supplementary Materials Supplementary Table 1: demographic and tumor characteristics of pancreatic cancer patients in high-, mid-, and low-risk groups. Supplementary Figure 1: ROC of postoperative complication prediction for the random forest, extreme gradient boosting, deep neural network, support vector machine, and logistic regression in training set and testing set. DeLong test: na, *P* value > 0.05; ∗, *P* value < 0.05; ∗∗, *P* value < 0.01; ∗∗∗, *P* value < 0.001. Supplementary Figure 2: (A) survival comparison between PC patients who receive chemotherapy and nonchemotherapy in the middle-risk group (after PSM). (B) Survival comparison between PC patients who receive chemotherapy and nonchemotherapy in the low-risk group (after PSM). (C) Survival comparison between PC patients who receive radiotherapy and nonradiotherapy in the low-risk group (after PSM).
