# Supplementary material for: Risk Assessment of Liver Metastasis in Pancreatic Cancer Patients Using Multiple Models Based on Machine Learning: A Large Population-Based Study
Source: Dis Markers. 2022 May 18;2022:1586074. doi: 10.1155/2022/1586074 (PMC9132665; doi:10.1155/2022/1586074)
Supplement: Supplementary Materials — Supplementary Table 1: demographic and tumor characteristics of pancreatic cancer patients in high-, mid-, and low-risk groups. Supplementary Figure 1: ROC of postoperative complication prediction for the random forest, extreme gradient boosting, deep neural network, support vector machine, and logistic regression in training set and testing set. DeLong test: na, P value > 0.05; ∗, P value < 0.05; ∗∗, P value < 0.01; ∗∗∗, P value < 0.001. Supplementary Figure 2: (A) survival comparison between PC patients who receive chemotherapy and nonchemotherapy in the middle-risk group (after PSM). (B) Survival comparison between PC patients who receive chemotherapy and nonchemotherapy in the low-risk group (after PSM). (C) Survival comparison between PC patients who receive radiotherapy and nonradiotherapy in the low-risk group (after PSM). [file 1586074.f1.docx]

**Supplementary Tables**

**Supplementary Table 1.** Demographic and tumor characteristics of pancreatic cancer patients in High-, Mid- and Low-risk group.

| Characteristic | Low-risk | Mid-risk | High-risk |
| --- | --- | --- | --- |
|  | n=15973 | n=15973 | n=15973 |
| Age at PC diagnosis, No. (%), years |  |  |  |
| 20-49 | 1341 ( 8.4) | 746 ( 4.7) | 1231 ( 7.7) |
| 50-69 | 8645 (54.1) | 6147 (38.5) | 9763 (61.1) |
| ≥ 70 | 5987 (37.5) | 9080 (56.8) | 4979 (31.2) |
| Sex, No. (%) |  |  |  |
| Female | 7913 (49.5) | 8489 (53.1) | 6722 (42.1) |
| Male | 8060 (50.5) | 7484 (46.9) | 9251 (57.9) |
| Year of PC diagnosis, No. (%) |  |  |  |
| 2010-2013 | 6568 (41.1) |  |  |
| 2014-2018 | 9405 (58.9) | 9721 (60.9) | 9910 (62.0) |
| Race, No. (%) |  |  |  |
| White | 12690 (79.4) | 12366 (77.4) | 12598 (78.9) |
| Black | 1793 (11.2) | 1984 (12.4) | 2250 (14.1) |
| Other | 1490 ( 9.3) | 1623 (10.2) | 1125 ( 7.0) |
| Primary tumor site, No. (%) |  |  |  |
| Head of pancreas | 10979 (68.7) | 10868 (68.0) | 4398 (27.5) |
| Body of pancreas | 1691 (10.6) | 1987 (12.4) | 3358 (21.0) |
| Tail of pancreas | 1795 (11.2) | 953 ( 6.0) | 4632 (29.0) |
| Overlapping lesion of pancreas | 1508 ( 9.4) | 2165 (13.6) | 3585 (22.4) |
| AJCC T stage, No. (%) |  |  |  |
| T1/T2 | 4534(28.4) | 4776(29.9) | 7907(49.5) |
| T3/T4 | 11439 (71.6) | 11197 (70.1) | 8066 (50.5) |
| AJCC N stage, No. (%) |  |  |  |
| N0 | 7878(49.3) | 11319(70.9) | 9409(58.9) |
| N1/N2 | 8095 (50.7) | 4654 (29.1) | 6564 (41.1 |
| Tumor histology, No. (%) |  |  |  |
| Adenocarcinomas | 9397(58.8) | 13410(84.0) | 13679(85.6) |
| Other | 6576 (41.2) | 2563 (16.0) | 2294 (14.4) |
| Tumor size, No. (%) |  |  |  |
| (0-2] | 2604 (16.3) | 1605 (10.0) | 586 ( 3.7) |
| (2-5] | 9186 (57.5) | 9022 (56.5) | 5906 (37.0) |
| 5< | 4183 (26.2) | 5346 (33.5) | 9481 (59.4) |
| Number of nodes examined, No. (%) |  |  |  |
| None | 3373(21.1) | 13912(87.1) | 15498(97.0) |
| One or more | 12600 (78.9) | 2061 (12.9) | 475 ( 3.0) |
| Liver metastasis, No. (%) |  |  |  |
| No | 15867(99.3) | 12075(75.6) | 4068(25.5) |
| Yes | 106 ( 0.7) | 3898 (24.4) | 11905 (74.5) |
| Surgery, No. (%) |  |  |  |
| No | 3167(19.8) | 14861(93.0) | 15890(99.5) |
| Yes | 12806 (80.2) | 1112 ( 7.0) | 83 ( 0.5) |
| Chemotherapy, No. (%) |  |  |  |
| No | 4855(30.4) | 7603(47.6) | 6282(39.3) |
| Yes | 11118 (69.6) | 8370 (52.4) | 9691 (60.7) |
| Radiation, No. (%) |  |  |  |
| No | 9278(58.1) | 14348(89.8) | 15934(97.9) |
| Yes | 6695 (41.9) | 1625 (10.2) | 329 ( 2.1) |

**NOTE:** All of the patients included in this study were divided into three groups based on the level of Random Forest risk scores.

**Abbreviations:** AJCC indicates American Joint Committee on Cancer; PC, pancreatic cancer.

**Supplementary Figures**


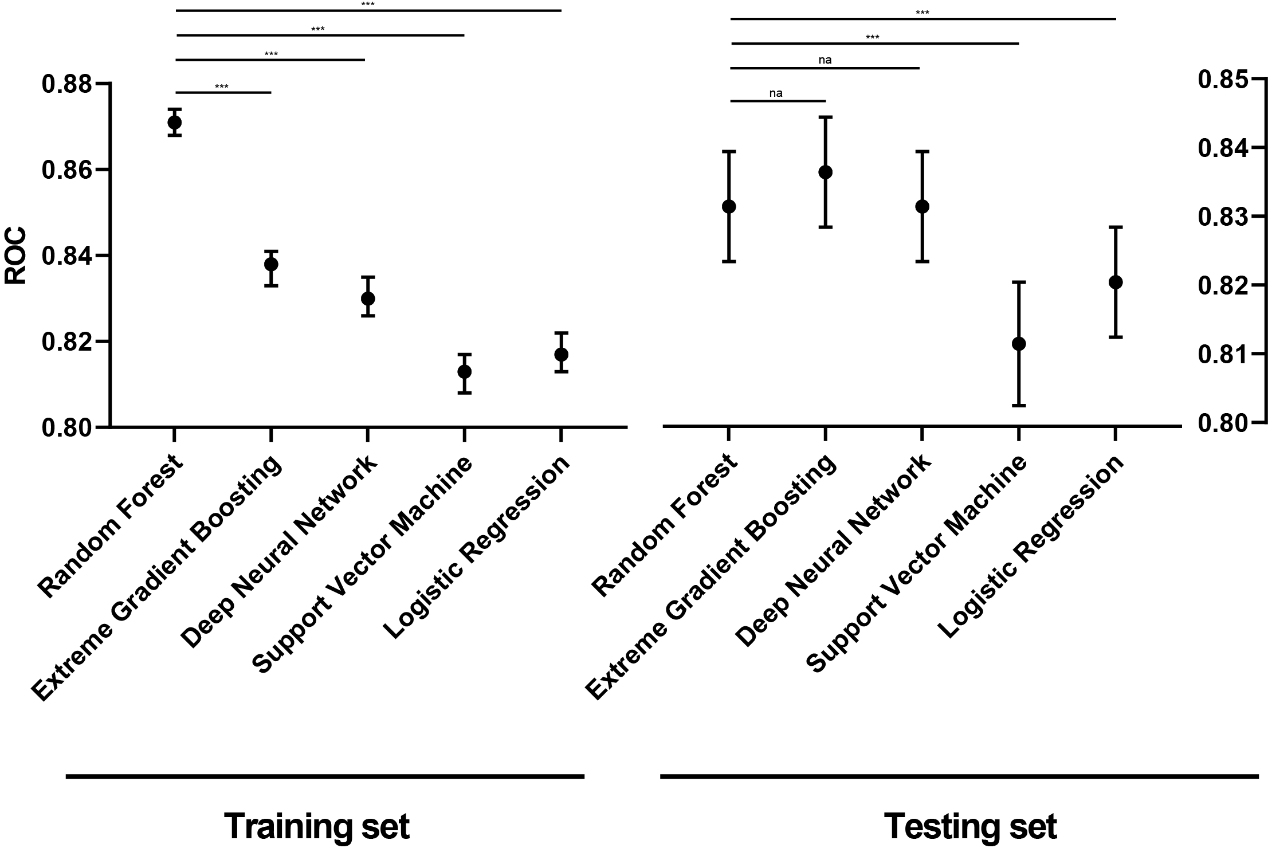


**Supplementary Figure 1.** ROC of postoperative complications prediction for the Random Forest, Extreme Gradient Boosting, Deep Neural Network, Support Vector Machine and Logistic Regression in training set and testing set. DeLong test; na, P-value>0.05; *, P-value <0.05; **, P-value <0.01; ***, P-value <0.001.


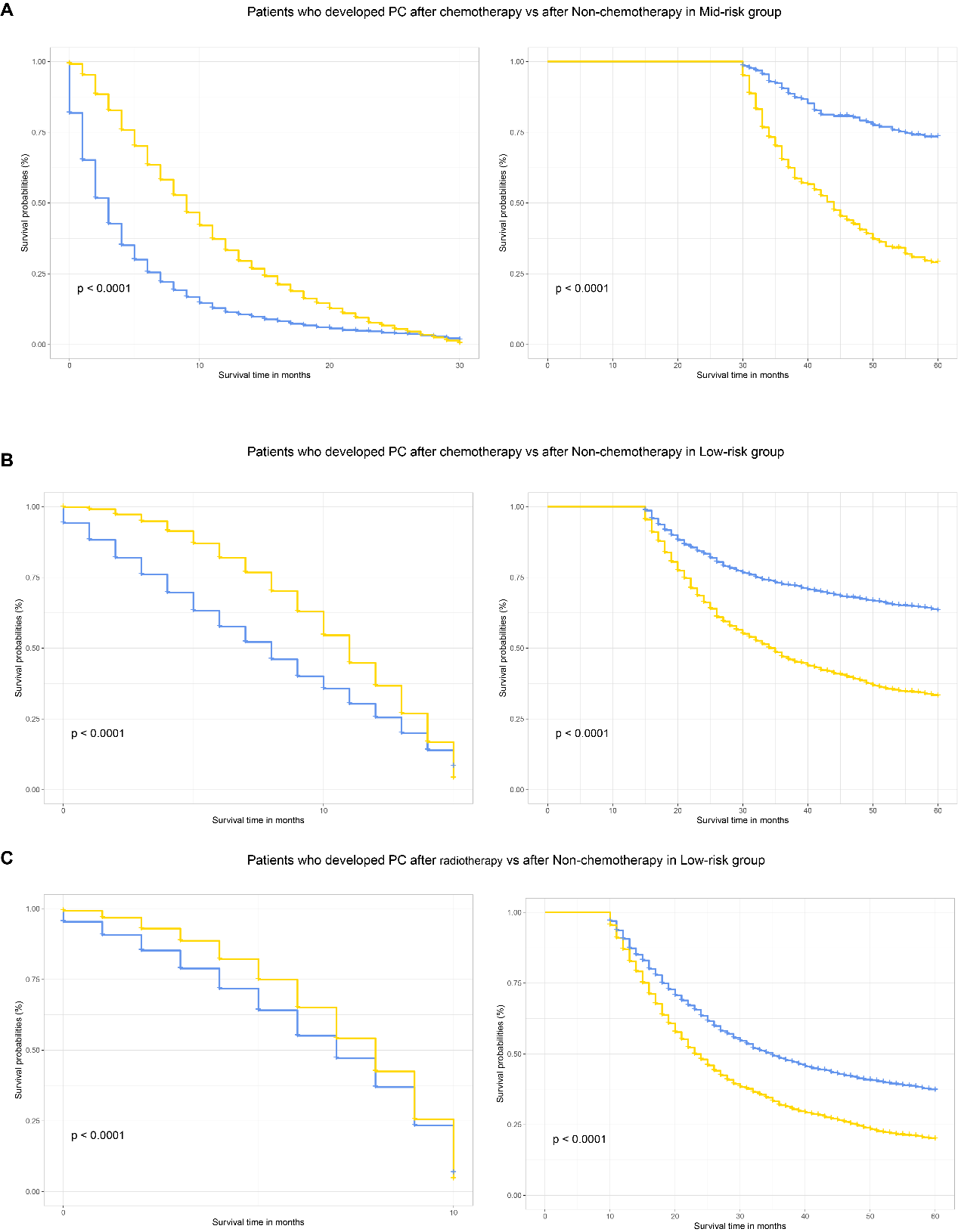


**Supplementary Figure 2.** (A) Survival comparison between PC patients who receive chemotherapy and non- chemotherapy in Middle -risk group (after PSM). (B) Survival comparison between PC patients who receive chemotherapy and non- chemotherapy in Low -risk group (after PSM). (C) Survival comparison between PC patients who receive radiotherapy and non- radiotherapy in Low -risk group (after PSM).
